# Supplementary material for: Bayesian refinement of protein structures and ensembles against SAXS data using molecular dynamics
Source: PLoS Comput Biol. 2017 Oct 18;13(10):e1005800. doi: 10.1371/journal.pcbi.1005800 (PMC5662244; doi:10.1371/journal.pcbi.1005800)
Supplement: S1 Archive — (BZ2) [file pcbi.1005800.s011.tar.bz2 › gromacs_4.62_bayesian_saxs_refi/share/html/online/g_rotacf.html]

g\_rotacf


| g\_rotacf Main Table of Contents |

**VERSION 4.6  
Sat 19 Jan 2013**

---

### Description

g\_rotacf calculates the rotational correlation function
for molecules. Atom triplets (i,j,k) must be given in the index
file, defining two vectors ij and jk. The rotational ACF
is calculated as the autocorrelation function of the vector
n = ij x jk, i.e. the cross product of the two vectors.
Since three atoms span a plane, the order of the three atoms
does not matter. Optionally, by invoking the -d switch, you can
calculate the rotational correlation function for linear molecules
by specifying atom pairs (i,j) in the index file.

EXAMPLES

g\_rotacf -P 1 -nparm 2 -fft -n index -o rotacf-x-P1
-fa expfit-x-P1 -beginfit 2.5 -endfit 20.0

This will calculate the rotational correlation function using a first
order Legendre polynomial of the angle of a vector defined by the index
file. The correlation function will be fitted from 2.5 ps until 20.0 ps
to a two-parameter exponential.

### Files

| option | filename | type | description |
| --- | --- | --- | --- |
| **-f** | traj.xtc | Input | Trajectory: xtc trr trj gro g96 pdb cpt |
| **-s** | topol.tpr | Input | Run input file: tpr tpb tpa |
| **-n** | index.ndx | Input | Index file |
| **-o** | rotacf.xvg | Output | xvgr/xmgr file |

### Other options

| option | type | default | description |
| --- | --- | --- | --- |
| **-[no]h** | bool | no | Print help info and quit || **-[no]version** | bool | no | Print version info and quit || **-nice** | int | 19 | Set the nicelevel || **-b** | time | 0 | First frame (ps) to read from trajectory || **-e** | time | 0 | Last frame (ps) to read from trajectory || **-dt** | time | 0 | Only use frame when t MOD dt = first time (ps) || **-[no]w** | bool | no | View output .xvg, .xpm, .eps and .pdb files || **-xvg** | enum | xmgrace | xvg plot formatting: xmgrace, xmgr or none || **-[no]d** | bool | no | Use index doublets (vectors) for correlation function instead of triplets (planes) || **-[no]aver** | bool | yes | Average over molecules || **-acflen** | int | -1 | Length of the ACF, default is half the number of frames || **-[no]normalize** | bool | yes | Normalize ACF || **-P** | enum | 0 | Order of Legendre polynomial for ACF (0 indicates none): 0, 1, 2 or 3 || **-fitfn** | enum | none | Fit function: none, exp, aexp, exp\_exp, vac, exp5, exp7, exp9 or erffit || **-ncskip** | int | 0 | Skip this many points in the output file of correlation functions || **-beginfit** | real | 0 | Time where to begin the exponential fit of the correlation function || **-endfit** | real | -1 | Time where to end the exponential fit of the correlation function, -1 is until the end |

---

http://www.gromacs.org  
gromacs@gromacs.org
